# Generalist host species drive *Trypanosoma cruzi* vector infection in oil palm plantations in the Orinoco region, Colombia

**DOI:** 10.1186/s13071-019-3519-3

**Published:** 2019-05-28

**Authors:** Diana Erazo, Nicole L. Gottdenker, Camila González, Felipe Guhl, Monica Cuellar, Troy J. Kieran, Travis C. Glenn, Juan D. Umaña, Juan Cordovez

**Affiliations:** 1 Grupo de Investigación en Biología Matemática y Computacional (BIOMAC), Universidad de los Andes, Bogota, Colombia; 2 Department of Pathology, School of Veterinary Medicine, The University of Georgia, Athens, GA USA; 3 Centro de Investigaciones en Microbiología y Parasitología Tropical (CIMPAT), Facultad de Ciencias, Universidad de los Andes, Bogotá, Colombia; 4 Grupo de Investigación en Ingeniería Biomédica (GIB), Universidad de los Andes, Bogota, Colombia; 5 Department of Environmental Health Science, College of Public Health, University of Georgia, Athens, GA USA

**Keywords:** *Trypanosoma cruzi*, *Rhodnius prolixus*, *Elaeis guineensis*, Blood meal analysis, Host community composition, Generalist host

## Abstract

**Background:**

Oil palm plantation establishment in Colombia has the potential to impact Chagas disease transmission by increasing the distribution range of *Rhodnius prolixus*. In fact, previous studies have reported *Trypanosoma cruzi* natural infection in *R. prolixus* captured in oil palms (*Elaeis guineensis*) in the Orinoco region, Colombia. The aim of this study is to understand *T. cruzi* infection in vectors in oil palm plantations relative to community composition and host dietary specialization by analyzing vector blood meals and comparing these results to vectors captured in a native palm tree species, *Attalea butyracea*.

**Methods:**

*Rhodnius prolixus* nymphs (*n* = 316) were collected from *A. butyracea* and *E. guineensis* palms in Tauramena, Casanare, Colombia. Vector blood meals from these nymphs were determined by amplifying and sequencing a vertebrate-specific *12S* rRNA gene fragment.

**Results:**

Eighteen vertebrate species were identified and pigs (*Sus scrofa*) made up the highest proportion of blood meals in both habitats, followed by house mouse (*Mus musculus*) and opossum (*Didelphis marsupialis*). Individual bugs feeding only from generalist mammal species had the highest predicted vector infection rate, suggesting that generalist mammalian species are more competent hosts for *T. cruzi* infection .

**Conclusions:**

Oil palm plantations and *A. butyracea* palms found in altered areas provide a similar quality habitat for *R. prolixus* populations in terms of blood meal availability. Both habitats showed similarities in vector infection rate and potential host species, representing a single *T. cruzi* transmission scenario at the introduced oil palm plantation and native *Attalea* palm interface.

**Electronic supplementary material:**

The online version of this article (10.1186/s13071-019-3519-3) contains supplementary material, which is available to authorized users.

## Background

Land-use change (LUC), caused by urbanization, agricultural expansion and intensification, leads to habitat fragmentation and loss of animal and plant biodiversity. Species responses to LUC are complex, potentially depending on their functional traits [1]. Typically in a community, large, slower breeding, less-mobile species as well as dietary and habitat specialists are the most vulnerable to LUC [2–8]. The specialist-generalist concept is based on how natural selection promotes specialized strategies among species by showing an evolutionary trade-off between specializing to perform few activities well, and generalizing to perform many activities fairly [9]. Specialists usually benefit from undisturbed landscapes, where there are many environmental niches in which to specialize, whereas generalists can often thrive in anthropogenically disturbed landscapes [10–13]. In fact, niche evolution theory predicts that habitat fragmentation should negatively affect specialist species in a community [14].

Recent studies in the tropics observe vertebrate specialist species declines as a consequence of LUC. Birds have been suggested as the most sensitive class negatively affected by forest conversion into agriculture around the world [15]. For instance, forest cover loss was the principle threat to specialist birds in the tropical rainforest of Lacandona in Mexico [16]. In general, mammals are less sensitive to habitat disturbance compared to birds, perhaps due to a higher abundance of generalist species, such as small mammals [15]. Nonetheless, anthropogenic intervention in tropical forests has caused a decrease in small mammal species that are forest specialists, favoring those that tolerate human activities in Costa Rica [17], Paraguay [18] and Venezuela [19]. Additionally, medium-sized opportunistic carnivores-omnivores, such as opossums and raccoons, tend to increase in abundance in disturbed landscapes [20, 21]. As a result, land use alteration is expected to decrease specialist vertebrate species, whereas generalist mammal species such as common opossum increases.

Generalist rodents, opossums, raccoons, and other opportunistic mammals are often considered to be important reservoirs for vector-borne diseases [22–25]. In many cases, these species are associated with rapid reproduction [26, 27], introducing high numbers of susceptible individuals into a population at a relatively high rate. In addition, based on the ‘pace of life’ hypothesis, fast-lived species are expected to invest less in acquired immunity compared to long-lived species [28–30]. Therefore, LUC favoring ‘fast-living’ generalist/opportunistic species could promote vector-borne disease transmission. Few studies have assessed the role of generalist species on vector-borne transmission. For instance, generalist rodents often harbor more diverse flea communities and higher flea loads (number of fleas per host) [31]. Another example from a modeling approach showed that dietary generalist species could amplify West Nile virus transmission compared to specialist species [32]. Clearly, the degree of habitat specialization of different host species should be considered when evaluating mechanisms of changes in vector-borne pathogen transmission in association with LUC.

Chagas disease is caused by a multi-host vector-borne pathogen and its transmission may be strongly affected by changes in host community as a consequence of LUC [33]. The disease is caused by the parasite *Trypanosoma cruzi* and transmitted by insects within the subfamily Triatominae. Chagas disease is endemic in Latin America affecting 7 million people, with a burden of 12,000 deaths per year [34]. The parasite transmission involves nearly a hundred mammal species, yielding domestic and sylvatic cycles of transmission [35]. Therefore, LUC, which alters mammal community composition, could potentially alter the disease transmission dynamics, requiring a particular understanding of the ecological transmission context for each LUC scenario [36].

A recent study in the Orinoco basin in Colombia showed that the mammal community in savannas and oil palm plantations (*Elaeis guineensis*) was similar, dominated by generalist species, and less rich compared to gallery forests [37]. In addition, *Rhodnius prolixus*, the main vector in the region, and a true palm specialist [38, 39], is capable of invading oil palm crowns, potentially introducing *T. cruzi* [40]. Thus, *T. cruzi* transmission is an interesting system for studying the role of dietary generalist versus specialist mammal host species in vector-borne disease transmission in agricultural landscapes, such as oil palm plantations, which are leading producers of biodiesel worldwide [41]. The rapid expansion of the oil palm industry in Colombia [42] could therefore have a significant impact on vector-host-*T.cruzi* transmission relationships.

The aim of this study was to understand the role of host community composition and the relative contribution of domestic and sylvatic mammal species on *T. cruzi* maintenance and transmission in oil palm plantations and adjacent native palm vegetation. This study has three specific objectives: (i) to analyze *R. prolixus* blood meals in oil palm plantations (African oil palms) in the Orinoco basin and investigate the importance of generalist host species in *T. cruzi* transmission in this agricultural landscape; (ii) to compare vector blood meals in oil palms with adjacent native *A. butyracea* palms, the natural habitat of *R. prolixus* in the region; and (iii) to determine if vector infection in both palm species is responding to similar drivers in terms of host dietary specialization.

## Methods

### Study area and triatomine sampling

Fieldwork was conducted in Los Potrillos, Tauramena municipality (4°59′1″N, 72°36′36″W) located in the department of Casanare, from August 2016 to July 2017. The region has bimodal seasonality [43] and we visited the study site twice per season, in August 2016 and July 2017 for the rainy season, and in December 2016 and March 2017 for the dry season. Each visit lasted 10 nights. We sampled a 2 ha *Attalea butyracea* forest and an adjacent *Elaeis guineensis* plantation (11 ha), for 5 consecutive nights per habitat.

Traps live baited with chickens were used for Triatomine collection [44]. Traps were set at 17:00 h within or next to the palm tree crowns, and revised the next day at 7:00 h. All available *A. butyracea* were sampled (*n* = 79) while for *E. guineensis* a subsample was selected based on the height of the crown suitable for sampling (*n* = 103) (see Additional file 1: Figure S1 for traps arrangement in the study site). Collected triatomines were placed in ethanol 70%. Palm trees were geo-referenced and marked for identification.

### Triatomine infection and blood meal analysis

We captured 316 *R. prolixus* nymphs (N1 to N5 nymphal stages) from the *E. guineensis* plantation (*n* = 148) and the *A. butyracea* forest (*n* = 168), to analyze for *T. cruzi* infection and blood meals. DNA was extracted using phenol:clorophorm:isoamyl alcohol protocol as described elsewhere [45].

*Trypanosoma cruzi* infection for selected insects was determined by the amplification of mini-circle specific primers 121 (5′-AAA TAA TGT ACG G(T/G)G AGA TGC ATG A-3′) and 122 (5′-GGG TTC GAT TGG GGT TGG TGT-3′) to obtain an amplicon of 330 bp [46]. DTU characterization was conducted by amplifying the intergenic region of the non-transcribed mini-exon gene from the parasite using primers TCC (5′-CCC CCC TCC CAG GCC ACA CTG-3′), TCI (5′-GTG TCC GCC ACC TCC TTC GGG CC-3′), and TC2 (5′-CCT GCA GGC ACA CGT GTG TGT G-3′) [47].

For blood meal analysis, we amplified DNA performing two PCR rounds following Kieran et al. [45]. The first-round PCR amplified a *12S* rRNA region (145 bp) typically used for vertebrate detection (F: 5′-CAA ACT GGG ATT AGA TAC C-3′; R: 5′-AGA ACA GGC TCC TCT AG-3′) [48] with TruSeq compatiable adaptor fusions [43]. PCR cycling conditions considered an initial denaturation at 98 °C for 3 min, followed by 40 cycles at 95 °C for 30 s, 63 °C for 1 min, 72 °C for 1 min, and a final extension at 72 °C for 5 min. Positive amplicons were pooled in equal concentration and cleaned with SPRI-beads (1:1 ratio).

For the second-round PCR we used Ilumina TruSeqHT compatible 8 nt indexed primers [49]. Reactions and thermocycler conditions were described by Kieran et al. [45]. We cleaned the library product and removed the primers using 1:1 ratio of SPRI-beads. Finally, the libraries were sent for sequencing on an Illumina MiSeq with Paired-End 300 reads (University of Georgia Genomics Facility).

Bioinformatics analyses were conducted using Mr Demuxy v1.2.0 (https://pypi.python.org/pypi/Mr_Demuxy/1.2.0), Geneious v10 (Biomatters Limited, NJ), and the software package QIIME v1.9.1 [50]. First, we demultiplexed the 12S amplicon pool using Mr Demuxy v1.2.0 to remove internal barcodes and primers. Then demultiplexed files were transferred to Geneious v10 to set paired-reads (size 145 bp) and trimmed for removing low quality bases (0.001 score). Finally, we imported data into QIIME v1.9.1 for assigning data Operational Taxonomic Unit (OTU), using UCLUST (similarity: 95%), based on a previously compiled *12S* reference database. For each sample, the OTU identification showed the total number of reads per species and those with less than 10% of total reads hits were removed.

The *12S* reference database consisted on the *12S* DNA sequences of vertebrate species reported in the Orinoco and its bordering regions (Amazonian and Andean) [51–54], downloaded from GenBank. When the complete *12S* sequence of a designated species was missing, we included an alternative sequence corresponding to a closely related species at a higher taxonomic level (genus or family). All reference sequences corresponded to the *12S* rRNA gene, 145 bp region. For reference database see Additional file 2 and for taxonomy see Additional file 3.

We did not include chicken (*Gallus gallus*) reads to account for contamination coming from the baited live traps used for triatomine capture. Additionally, we performed an additional PCR to samples reporting human reads to determine contamination by amplifying the human beta globin gene (268 bp) using primers GH20/PC04 [55]. The human beta globin gene amplification is commonly used as a control for human clinical samples subject to molecular analysis [56]. In addition, this set of primers (GH20/PC04) has been used by other vector blood meal studies to confirm presence/absence of contamination with human DNA [57].

For data visualization, we developed an interaction network for each habitat where nodes represented *R. prolixus* and identified vertebrate species (OTUs). Links are unidirectional and move from every identified vertebrate species to vector, because they provide food for *R. prolixus.* Link weights were determined separately for each habitat, by normalizing the number of blood meals of each identified species. Therefore, the link weight between the vertebrate species presenting the highest number of blood meals in a particular habitat and *R. prolixus* is 1.

### Data analysis

A Chi-square test of independence was used to measure the dependence between taxonomic class, taxonomic order, species identified in vector blood meals, and habitat type. A generalized linear model with binomial error distribution was used to test the effects of most abundant mammal species on *T. cruzi* infection in *R. prolixus.* A Pearson correlation was used to test the relationship between host body size and infection in *R. prolixus,* for the study site and discriminated by habitat. Skin surface area has been suggested as a proper way to reflect host body size. However, such data are not available, thus we used the allometric scaling relationship between body mass *M* and skin surface *A* given by the expression *A* α *M*^*2/3*^ [58]. Body mass and size for each host species are provided in Additional file 1: Table S1. When more than one host species was found in the a single vector blood meal, we used the average body size.

We used regression tree models to evaluate the relationship between vector infection prevalence and habitat associations of identified potential host species (domestic and sylvatic species). We divided sylvatic species into two groups, ecological specialist and generalist species based on feeding preferences [59]. Only omnivores were considered as true generalist species (see Additional file 1: Table S1). Presence/absence of identified potential host species was the explanatory variable, thus the detection of any ecological species (domestic, specialist, and generalist) in an individual would be binomial (1 for presence and 0 for absence) for a specific category. Finally, we computed Moran’s *I* index for determining spatial aggregation in the abundance of blood meal species identified in *R. prolixus*. Moran’s *I* gives values ranging from − 1 to + 1. *I* equals zero when there is no spatial autocorrelation (H_0_), negative when there is negative autocorrelation and positive when data presents a clustering pattern.

We visualized *R. prolixus* and identified vertebrate species (OTUs) networks using the interactive platform Gephi [60]. One network is depicted per habitat, in which links are unidirectional from identified vertebrate species, that provide blood meal, to the vector. Link weights are specific and normalized for each habitat. We used R version 3.3.2 [61] and the RStudio Integrated Development Environment (IDE) for the analyses. Statistical analysis was conducted using the *base* package. We used the *rpart* package to perform the regression tree models [62]. For computing the Moran’s I index we used the *ape* package [63].

## Results

### Triatomine infection and blood meals

Triatomine infection rate was similar between the two habitats: 75% in the oil palm plantation (111/148) and 76% in the *A. butyracea* forest (125/165). The obtained DTU in 22 examined *R. prolixus* individuals corresponded to TcI.

We did not detect human beta globin gene in any of the processed samples, suggesting contamination in steps performed before *12S* amplification (insect collection and/or DNA extraction), so no human blood source was considered in the study.

**Fig. 1:**
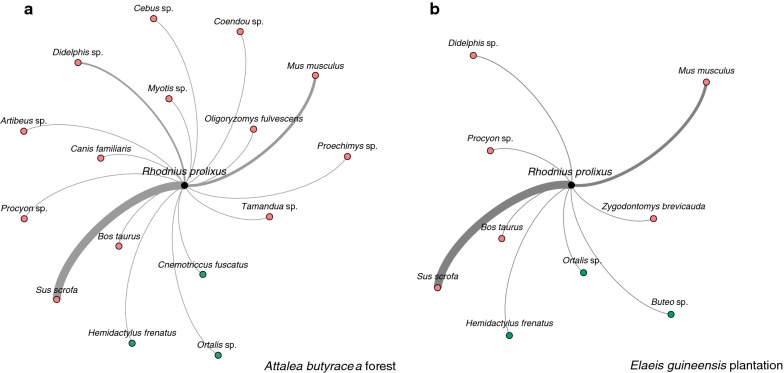
The interaction networks for *Rhodnius prolixus* blood meals the *A. butyracea* forest and the *E. guineensis* plantation. Nodes represent *R. prolixus* and vertebrate species. The vector node is colored in black, mammal nodes are pink colored, bird and reptile nodes are green colored. We considered unidirectional links, colored in gray, from every vertebrate species that provides a blood meal to the vector. Links vary in strength depending on the number of vector blood meals identified from each vertebrate species in a particular habitat. To determine link strength we normalized each blood meal species identified in *R. prolixus*. Therefore the link weight between the vector and the vertebrate species showing the highest number of blood meals is 1, which in both habitats is *Sus scrofa*, followed by *Mus musculus* and *Didelphis* sp

We detected vertebrate DNA in 94.6% of *R. prolixus* samples (*n* = 299). Blood meal analysis detected a total of 18 vertebrate species, consisting of 14 mammals, 3 birds, and 1 reptile (Fig. 1). Mammals comprised 95.9% of vector blood meals, while birds and reptiles constituted 3.4% and 0.7%, respectively. There was no significant association between taxonomic class and habitat type (*χ*^2^ = 1.75, *df* = 2, *P* = 0.41). The pig (*Sus scrofa*) made up the highest proportion of blood meals with 59.2%, followed by the house mouse (*Mus musculus*) (19.6%), and opossum (*Didelphis sp*) (8.7%). This ranking pattern was maintained in the *A. butyracea* forest and the oil palm plantation (Tables 1, 2).

**Table 1:** Blood meal species detected in *R. prolixus* individuals collected in the *A. butyracea* forest

Class	Order	Species	Reads	% Reads^a^	Blood meals	% Blood meals	Weight
Aves	Galliformes	*Ortalis* sp.	15,946	2.11	8	3.5	0.06
	Passeriformes	*Cnemotriccus fuscatus*	4125	0.55	2	0.9	0.02
Reptilia	Squamata	*Hemidactylus frenatus*	9583	1.27	2	0.9	0.02
Mammalia	Artiodactyla	*Bos taurus*	963	0.13	2	0.9	0.02
		*Sus scrofa*	247,319	32.71	124	54.4	1.00
	Carnivora	*Canis lupus familiaris*	4073	0.54	4	1.8	0.03
		*Procyon* sp.	5391	0.71	6	2.6	0.05
	Chiroptera	*Artibeus* sp.	6837	0.90	2	0.9	0.02
		*Myotis* sp.	75,280	9.96	9	3.9	0.07
	Marsupialia	*Didelphis* sp.	134,197	17.75	25	11.0	0.20
	Primata	*Cebus* sp.	195	0.03	1	0.4	0.01
	Rodentia	*Coendou* sp.	143	0.02	1	0.4	0.01
		*Mus musculus*	211,141	27.92	36	15.8	0.29
		*Oligoryzomys fulvescens*	25,832	3.42	2	0.9	0.02
		*Proechimys* sp.	1602	0.21	2	0.9	0.02
	Xenarthra	*Tamandua* sp.	13,507	1.79	2	0.9	0.02
		Unassigned	141,853		–	–	–

^a^Percentage after removing unassigned reads

**Table 2:** Blood meal species detected in *R. prolixus* individuals collected in the *E. guineensis* plantation

Class	Order	Species	Reads	% Reads^a^	Blood meals	% Blood meals	Weight
Aves	Accipitriformes	*Buteo* sp.	2543	0.58	1	0.5	0.01
	Galliformes	*Ortalis* sp.	1391	0.32	3	1.6	0.02
Reptilia	Squamata	*Hemidactylus frenatus*	6042	1.37	1	0.5	0.01
Mammalia	Artiodactyla	*Bos taurus*	674	0.15	1	0.5	0.01
		*Sus scrofa*	232,321	52.70	121	65.1	1.00
	Carnivora	*Procyon* sp.	1176	0.27	2	1.1	0.02
	Marsupialia	*Didelphis* sp.	33032	7.49	11	5.9	0.09
	Rodentia	*Mus musculus*	160304	36.37	45	24.2	0.37
		*Zygodontomys brevicauda*	3313	0.75	1	0.5	0.01
		Unassigned	133615	–	–	–	–

^a^Percentage after removing unassigned reads

*Rhodnius prolixus* from both habitats shared blood meals of four mammalian orders: Artiodactyla, Carnivora, Marsupialia and Rodentia. Three additional mammal orders were identified in the *A. butyracea* forest, Chiroptera (5%), Primates (1%) and Xenarthra (1%) (Tables 1, 2). There was a significant association between taxonomic order and habitat type (*χ*^2^ = 22.2, *df* = 6, *P* = 0.001). Species composition varied between habitats. The *A. butyracea* forest had more diverse blood meal sources (16 species) compared to the *E. guineensis* plantation (9 species), and the association between species and habitat type was significant (*χ*^2^ = 29.9, *df* = 13, *P* = 0.005). However, when considering solely shared identified species (*Bos taurus*, *Didelphis* sp., *M. musculus, Procyon* sp. and *Sus scrofa*), which were the great majority in both habitats (*A. butyracea*: 85% and *E. guineensis*: 97%), the association was not significant (*χ*^2^ = 8.37, *df* = 4, *P* = 0.08).

We found *R. prolixus* individuals with multiple blood meals (Table 3). Double species were detected in 26.6% (*n* = 84) and triple species were detected in 4.7% (*n* = 15), although 63.3% of the samples had a single blood meal (*n* = 200).

**Table 3:** Percentage (number) of blood meal species identified per vector discriminated by stage and habitat

Stage	*Attalea butyracea* forest					*Elaeis guineensis* plantation				
	0	1	2	3	Total	0	1	2	3	Total
N1	0	0.6 (2)	0.3 (1)	0	0.9 (3)	0	0.6 (2)	0.3 (1)	0	0.9 (3)
N2	0.3 (1)	4.4 (14)	1.9 (6)	0	6.6 (21)	0.3 (1)	4.4 (14)	0.6 (2)	0	5.4 (17)
N3	0.6 (2)	7.9 (25)	2.2 (7)	1.3 (4)	12 (38)	0.9 (3)	10.1 (32)	4.7 (15)	0.6 (2)	16.5 (52)
N4	0.3 (1)	5.7 (18)	4.7 (15)	0.3 (1)	11.1 (35)	0.9 (3)	7.9 (25)	10.1 (15)	0.3 (1)	13.9 (44)
N5	0.9 (3)	14.9 (47)	4.4 (14)	2.2 (7)	22.5 (71)	0.9 (3)	6.6 (21)	2.5 (8)	0	10.1 (32)
Total	2.2 (7)	33.5 (106)	13.6 (43)	3.8 (12)	53.2 (168)	3.2 (10)	29.7 (94)	13 (41)	0.3 (1)	46.8 (148)

### Blood meals and *T. cruzi* infection

**Fig. 2:**
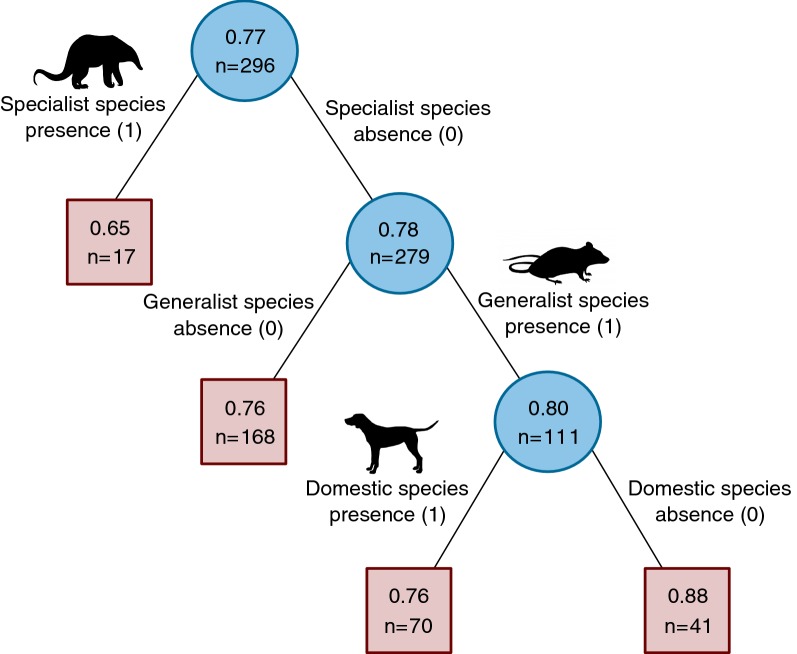
Regression tree analysis for vector infection. To test the relationship between vector infection prevalence and host species habitat associations we used a regression tree model. We considered domestic, generalist, and specialist sylvatic as habitat associations. Circles and rectangles represent non-terminal and terminal nodes, respectively. The circled node at the top is labeled with the number of infected individuals (*n* = 296) and total *R. prolixus* infection rate variance explained by the tree (77%). Links between nodes are labeled with the presence (1) or absence (0) of a species-habitat association. All nodes are labeled with the predicted infection rate and the number of individuals that meet the preceding link(s) condition(s). The highest infection rate, 88%, was predicted for *R. prolixus* feeding solely from generalist mammal species. On the other hand, the lowest infection rate (64%) was predicted for individuals having blood meals only from specialist mammal species

The regression tree analysis shown in Fig. 2 has *T. cruzi* infection in vectors as response variable and domestic, generalist, and specialist sylvatic mammal species presence as explanatory variables. In the tree non-terminal and terminal nodes are illustrated as circles and rectangles, respectively. The main node at the top of the tree shows the number of individuals evaluated for this tree and subsequent nodes are labeled with the predicted infection rate and the number of individuals that corresponded to the node. Links between nodes are labeled with an explanatory variable, showing its value (1 for presence and 0 for absence).

The regression tree explained 77% of the total variance in the response variable. Predicted infection rate was the lowest for individuals feeding exclusively from specialist mammal species, such as *Cebus* sp., bats (*Artibeus* sp. and *Myotis* sp.), specialist rodents (*Proechimys* sp. and *Coendou* sp.) and anteater (*Tamandua* sp.). The highest infection was predicted for individuals feeding only from generalist or opportunistic mammal species: opossum, raccoon (*Procyon* sp.), house mouse, and the generalist rodents, *Oligoryzomys fulvescens* and *Zygodontomys brevicauda*. For individuals feeding from domestic and generalist mammals, the regression tree predicted an infection rate of 76%. We did not find vectors feeding from both domestic and sylvatic species.

The general linear model showed no significant effect of *S. scrofa*, *Didelphis* sp., nor *M. musculus* blood meals on vector *T. cruzi* infection. In this analysis we considered single, dual, and triple blood meals that detected these species. The Pearsonʼs correlation test showed no relation between host body size and vector infection for the study site (*r* = 0.0018, *P* = 0.97), forest (*r* = -0.1302, *P* = 0.13), or plantation (*r* = 0.1080 *P* = 0.18).

### Blood meals spatial patterns

**Fig. 3:**
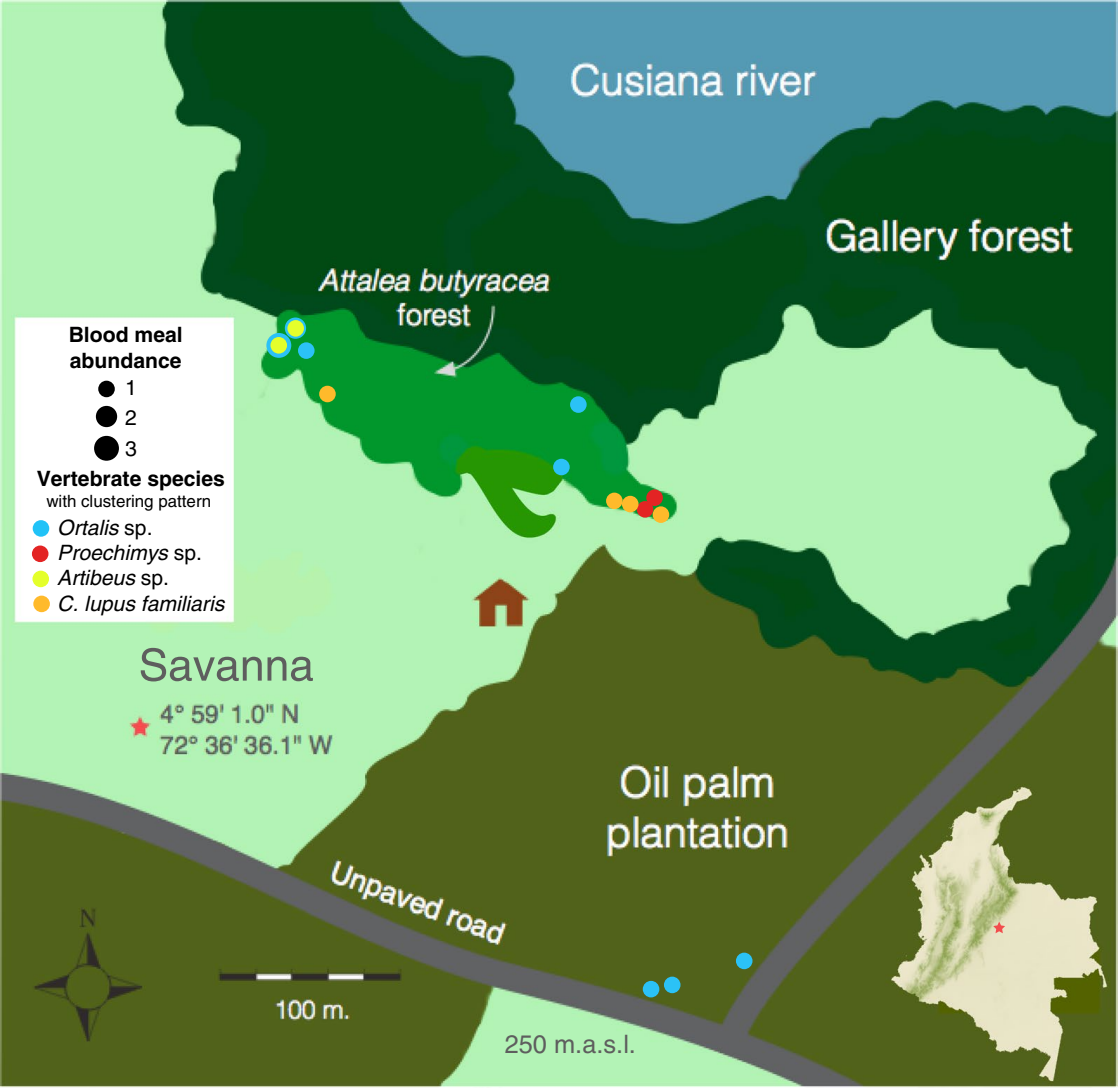
Spatial distribution of clustered vector blood meals. Los Potrillos, Tauramena is located in the department of Casanare (Orinoco region, Colombia). The study site is adjacent to the Cusiana River and covers an area of 25 ha that comprehends savanna, *A. butyracea* forest, gallery forest, and oil palm plantation landscapes. Additionally, peridomestic areas are present. Vectors were collected in *A. butyracea* and *E. guineensis* palms. Based on Moran’s *I* index computed for the abundance of blood meal species identified in *R. prolixus*, most blood meals were randomly distributed in the study area. The few species that exhibited clustering were *Ortalis* sp. (blue), *Proechimys* sp. (red), *Artibeus sp* (yellow), and *C. lupus familiaris* (orange)

Few species identified in the blood meals had significant Moran’s *I* indices different from zero (*P < *0.1), implying that a great majority of blood meals were randomly distributed in the study site. We found positive significant *I* indices for *Ortalis* sp. (*I* = 0.09, *P < *0.05), *Proechimys* (*I* = 0.03, *P* = 0.004), *Artibeus* sp. (*I* = 0.09, *P < *0.05), and *Canis lupus familiaris* (*I* = 0.02, *P* = 0.07), thus these species blood meals displayed a clustering pattern (Fig. 3).

## Discussion

Palm trees are the natural ecotope of most *Rhodnius* species [39] because they provide refuge and plenty of food sources, as many vertebrate species forage there [64–66]. *Attalea butyracea* is a complex-crown palm tree that is ubiquitous in the Orinoco region, where large *R. prolixus* densities with high *T. cruzi* natural infection rates [67–69] and blood meals from all terrestrial vertebrate taxa, except amphibians, have been reported [70]. In this study, we present a detailed description and analysis of *R. prolixus* blood meals in *A. butyracea* palms and oil palms (*E. guineensis*) in the department of Casanare, Colombia.

Oil palm plantations have been suggested as a new ecotope for *R. prolixus* and *T. cruzi* maintenance [40]. Here, our findings suggest that *E. guineensis* palm trees could be providing *R. prolixus* and *T. cruzi* a habitat of similar quality to *A. butyracea* palm trees located in disturbed landscapes. In both habitats, vertebrate species identified from *R. prolixus* blood meals are similar, with minor differences in composition, providing a comparable enzoonotic scenario.

The Orinoco region is known as a high transmission area for Chagas disease. Reports showed a *T. cruzi* natural infection between 60–85% [68, 69] and a palm infestation index reaching 100% [71]. Here we showed high infection rates, comparable to previous studies, 76% in the *A. butyracea* palm trees and 75% in the *E. guineensis* plantation. Therefore, our finding that 95.6% of vector blood meals corresponded to mammalian hosts could be a possible explanation for such a high vector infection, because mammals are the only competent reservoirs for *T. cruzi* infection. The remaining blood meals were identified as coming from birds and geckos which are known to be refractory to *T. cruzi* infection [72, 73].

In this study, the domestic pig was the top ranked blood meal in both habitats, which was initially surprising, but is supported by the presence of a pigpen in the *A. butyracea* area. In addition, *Sus scrofa domestica* has been suggested as an attractive host species [74, 75]. Nonetheless, the spatial analysis on vectors displaying pig blood meal did not show a clustering pattern; instead these vectors were randomly distributed, probably because the pigpen is less than 200 meters far from the remotest palm tree in the forest. The spatial pattern of pig blood meals in the plantation could be attributable to the occasional release of pigs within the habitat area. Thus, our findings suggests that in this area, the pig could be a relevant food source for *R. prolixus*.

Regression tree analysis showed that generalist or opportunistic host species could be playing an important role in *T. cruzi* maintenance in the area. Habitat-generalist species are known for their synanthropic behavior, because they benefit from living in close proximity to humans. Thus, these species provide food source for triatomines in disturbed landscapes and are parasite reservoirs [23, 75]. Generalist rodents were detected in vector blood meals from both habitats and *M. musculus* was the predominant species. *Mus musculus* is an introduced Muridae species that has adapted to domestic and peridomestic habitats in many regions of Colombia [76], and its role as a *T. cruzi* competent reservoir needs to be tested. In a previous study, we found that 1 out of 12 individuals infected (8.3%) (unpublished data, Casanare, Colombia) and in Mexico 6.2% were *T. cruzi*-positive [77]. The common opossum, *D. marsupialis*, a well-known *T. cruzi* reservoir [22], showed an infection of 29% in this site (unpublished data), and comprised a significant proportion of vector blood meals in the *A. butyracea* forest and to a lesser extent, the *E. guineensis* plantation. Other identified generalist species were the crab-eating raccoon (*Procyon* sp.) and the rodents *O. fulvescens* and *Z. brevicauda*.

Blood meals from specialist species were absent in the oil palm plantation and present in a few bugs captured in the *A. butyracea* forest (7.5%), which is expected considering that the entire study site is particularly altered. Since specialist species tend to be more K-selected (lower reproductive rates and longer life spans) compared to opportunistic species [78], high vector infection rates in the area could also be explained by the little presence of these species in *R. prolixus* blood meals [33, 79].

We exclusively analyzed nymphs and therefore were expecting to identify mostly arboreal or scansorial vertebrate species. However, we found terrestrial species in nymph blood meals. This observation was also reported by Gottdenker et al. [33], where dog, pig, and cow were identified in vector blood meals. To our knowledge, these results could suggest two possibilities that are not mutually exclusive. Nymphs might be descending to the ground to feed and return to the palm trees, as suggested by previous reports highlighting the considerable power of triatomine dispersal [80]. Both *R. prolixus* nymphs and adults seem to migrate from their colonies to other sites by crawling [81] and/or passive-dispersal mechanisms, such as bugs clinging to birds [82]. The other possibility is that nymphs could be feeding from engorged adults who have fed from a terrestrial mammal before. This phenomenon known as hematoklepty [83–86] is also supported by multiple blood meals detection in first and second nymphal stage individuals in the present and a previous study [45]. However, recent reports suggest that thermal stimulation is the only cue triggering bites to conspecifics in *R. prolixus* and kissing bugs thermoregulate even when feeding on vertebrates [87]. Thus, the mechanism(s) explaining how early stage triatomines that are palm-associated feed from terrestrial species requires further study.

Previous reports in Colombia have detected vertebrate species in triatomine blood meals using ELISA and the *cytochrome b* gene PCR-HRM (polymerase chain reaction - high resolution melting); however, these studies identified fewer species than our study and higher taxonomic levels of sylvatic animals were not specified (bats and mice) [70, 88, 89]. For the ELISA approach, the fact that some species-specific anti-sera might not be available, could lead to unidentified species [88]. On the other hand, PCR-HRM protocol is available only for some species, which implies further standardization and required sequencing for not available species. Based on our results, we highly recommend using next-generation sequencing (NGS) method, which is an affordable and accurate method for identifying multiple triatomine blood meals in a single individual [45]. Detecting additional vertebrate species in vector blood meals besides easily identifiable species could help target novel host species and allow for a better understanding of triatomine ecology. This is particularly important, considering how land-use change may result in a host preference or availability switch [90, 91].

In this study, all processed nymphs were engorged at the time of collection, though chicken reads were present in a considerable number of samples, We do not discard the possibility of vectors feeding from chickens, which were highly present in the study site; however, given the study purposes, baited live traps with chickens could be a confounding element.

On the other hand, we obtained 18.7% of unassigned reads, suggesting gaps in the reference material. Currently, the Orinoco region still lacks biodiversity data for their species description and genetic database, partially due to previous inaccessibility to natural ecosystems as result of safety and security measures in this conflict-prone zone [92]. Finally, research on reservoir competence regarding the identified mammal species is necessary to fully understand *T. cruzi* enzoonotic transmission cycle in these habitats.

## Conclusions

We conclude that generalist host species, rather than specialist, could be driving high vector infection rates with *T. cruzi* in oil palm plantations in the study area. Additionally, we suggest that oil palm plantations in the Orinoco region could serve as an extension of highly altered habitats with *A. butyracea* palms in terms of reservoir host movement and *T. cruzi* transmission. This could be the case for *E. guineensis* plantations near highly intervened areas, such as peridomicile and cattle pasture. Nonetheless, further research is required to understand parasite transmission scenarios in plantations with different spatial configurations in relation to peridomicile and other habitats.

## Additional files

**Additional file 1: Figure S1.** Map of the study site. Triatomines were collected an *Attalea butyracea* forest (2 ha) and an oil palm plantation (11 ha). Black dots represent baited live traps locations in both habitats. **Table S1.** Data from mammal species found in vector blood meals, which were used for the statistical analyses.

**Additional file 2.** *12S* DNA reference database of vertebrate species from GenBank.

**Additional file 3.** *12S* DNA taxonomy file of vertebrate species from GenBank.

## Data Availability

Data supporting the conclusions of this article are included within the article and its additional files.

## Abbreviations

ANLA: Agencia Nacional de Licencias Ambientales-National Authority of Environmental Licenses
DTU: discrete taxonomic unit
ELISA: enzyme-linked immunosorbent assay
LUC: land use change
N1: first nymphal stage
N2: second nymphal stage
N3: third nymphal stage
N4: fourth nymphal stage
N5: fifth nymphal stage
NGS: next-generation sequencing
OTU: operational taxonomic unit
PCR: polymerase chain reaction
PCR-HRM: polymerase chain reaction-high resolution melting
