# Supplementary material for: Generalist host species drive Trypanosoma cruzi vector infection in oil palm plantations in the Orinoco region, Colombia
Source: Parasit Vectors. 2019 May 28;12:274. doi: 10.1186/s13071-019-3519-3 (PMC6540391; doi:10.1186/s13071-019-3519-3)
Supplement: Supplementary file 1 — Additional file 1: Figure S1. Map of the study site. Triatomines were collected an Attalea butyracea forest (2 ha) and an oil palm plantation (11 ha). Black dots represent baited live traps locations in both habitats. Table S1. Data from mammal species found in vector blood meals, which were used for the statistical analyses. [file 13071_2019_3519_MOESM1_ESM.docx]

**Additional file 1**

**Additional file 1: Figure S1.** The study site is located in the Municipality of Tauramena, Casanare (Colombia), which is characterized by savannas and gallery forests adjacent to water bodies. In this region oil palm plantations have mainly substituted savannas that previously were used for cattle and agriculture activities. The study site is adjacent to the Cusiana River and has an area of 25 ha. Here we collected triatomines in an *Attalea butyracea* forest (2 ha) and an oil palm plantation (11 ha). Black dots in the figure represent baited live traps locations in both habitats.

**
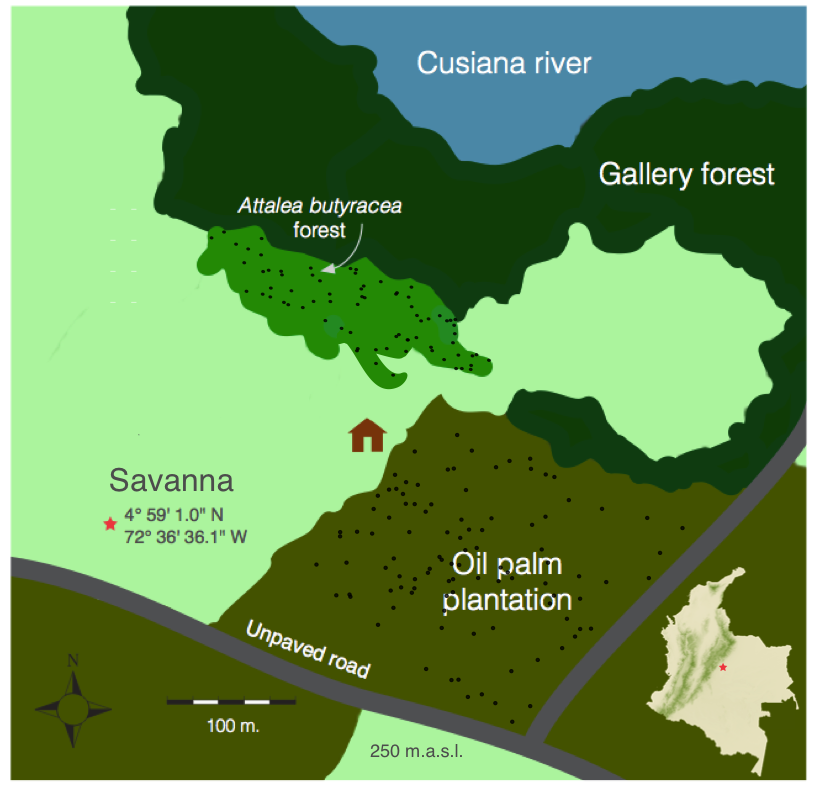
**

**Additional file 1: Table S1.** Data from mammal species found in vector blood meals, which was used for the statistical analyses. Body size was estimated by the calculation of skin surface area, using the allometric scaling relationship between body mass *M* and skin surface *A* given by the expression *A* α *M^2/3^*. Host body mass data was taken from (Myers et al., 2017; Parr et al., 2014).

| Order | Species | Diet | Group | Mass (grams) | Body size | References |
| --- | --- | --- | --- | --- | --- | --- |
| Artiodactyla | *Bos taurus* |  | Domestic | 347,000 | 4938 |  |
|  | *Sus scrofa* |  | Domestic | 150,000 | 2823 |  |
| Carnivora | *Canis lupus familiaris* |  | Domestic | 16,800 | 656 |  |
|  | *Procyon sp* | Omnivore | Generalist | 1,160 | 110 | (Francisco and Bisbal, 1986) |
| Chiroptera | *Artibeus sp* | Herbivore | Specialist | 66 | 16 | (Morrison, 1980) |
|  | *Myotis sp* | Insectivorous | Specialist | 12 | 5 | (Kalko et al., 1996) |
| Marsupialia | *Didelphis sp* | Omnivore | Generalist | 1,530 | 133 | (Emmons, 1990) |
| Primata | *Cebus sp* | Frugivore | Specialist | 2,200 | 169 | (Emmons, 1990) |
| Rodentia | *Coendou sp* | Herbivore | Specialist | 3,280 | 221 | (Roberts et al., 1985) |
|  | *Mus musculus* | Omnivore | Generalist | 23 | 8 | (Emmons, 1990) |
|  | *Oligoryzomys fulvescens* | Omnivore | Generalist | 15 | 6 | (Emmons, 1990; Utrera et al., 2000) |
|  | *Proechimys sp* | Frugivorous/  granivorous | Specialist | 23 | 8 | (Adler, 1995) |
|  | *Zygodontomys brevicauda* | Omnivore | Generalist | 22 | 8 | (Emmons, 1990; Utrera et al., 2000) |
| Xenarthra | *Tamandua sp* | Ants | Specialist | 3,500 | 231 | (Emmons, 1990) |

**Bibliography**

Adler, G.H., 1995. Fruit and Seed Exploitation by Central American Spiny Rats, Proechimys semispinosus. Stud. Neotrop. Fauna Environ. 30, 237–244. https://doi.org/10.1080/01650529509360962

Emmons, L., 1990. Neotropical Rainforest Mammals. A field guide. Chicago and London: The University of Chicago Press.

Francisco, J., Bisbal, E., 1986. Food habits of some neotropical carnivores in Venezuela (Mammalia, Carnivora). Mammalia 50, 329–340. https://doi.org/10.1515/mamm.1986.50.3.329

Kalko, E.K.V., Handley, C.O., Handley, D., 1996. Organization, Diversity, and Long-Term Dynamics of a Neotropical Bat Community, in: Long-Term Studies of Vertebrate Communities. pp. 503–553. https://doi.org/10.1016/B978-012178075-3/50017-9

Morrison, D.W., 1980. Foraging and Day-Roosting Dynamics of Canopy Fruit Bats in Panama. J. Mammal. 61, 20–29. https://doi.org/10.2307/1379953

Myers, P., Espinosa, C., Parr, T., Jones, G., Hammond, S., Dewey, T., 2017. The Animal Diversity Web (online). Univ. Michigan. URL http://animaldiversity.org

Parr, C.S., Wilson, N., Leary, P., Schulz, K., Lans, K., Walley, L., Hammock, J., Goddard, A., Rice, J., Studer, M., Holmes, J., Corrigan, Jr., R., 2014. The Encyclopedia of Life v2: Providing Global Access to Knowledge About Life on Earth. Biodivers. Data J. 2, e1079. https://doi.org/10.3897/BDJ.2.e1079

Roberts, M., Brand, S., Maliniak, E., 1985. The Biology of Captive Prehensile-Tailed Porcupines , Coendou prehensilis. Am. Soc. Mammal. 66, 476–482. https://doi.org/10.2307/1380922

Utrera, A., Duno, G., Ellis, B.A., Salas, R.A., de Manzione, N., Fulhorst, C.F., Tesh, R.B., Mills, J.N., 2000. Small mammals in agriculturalareas of the western llanos of Venezuela: community structure, habitat associations, and relative densities. J. Mammal. https://doi.org/10.1644/
